# Evaluating the Efficacy of Probiotics on Inflammatory Cytokines in Alcoholic Liver Disease: A Focus on IL-6 and IL-10

**DOI:** 10.3390/nu18040666

**Published:** 2026-02-18

**Authors:** Jiadila Bahetiyaer, Jie Cui, Wenhui Li, Jian Zhang, Ye Sun, Chunqing Ai, Shuying Liu, Shuaiming Jiang, Chengcheng Zhang, Jinchi Jiang

**Affiliations:** 1 State Key Laboratory of Marine Food Processing & Safety Control, Qingdao 266400, China; 17366104413@163.com (J.B.); acqdongying@163.com (C.A.); shuying.liu@bmsg.com (S.L.); 2 College of Biological and Pharmaceutical Engineering, Nanjing Tech University, Nanjing 211816, China; 3 College of Food Science and Light Industry, Nanjing Tech University, Nanjing 211816, China; jiecui_nitech@163.com (J.C.); lwhnjtec@163.com (W.L.); four-leaf-clover@njtech.edu.cn (J.Z.); sunye@nitech.edu.cn (Y.S.); 4 Key Laboratory of Food Nutrition and Functional Food of Hainan Province, School of Food Science and Engineering, Hainan University, Haikou 570228, China; 5 State Key Laboratory of Food Science and Resources, School of Food Science and Technology, Jiangnan University, Wuxi 214122, China

**Keywords:** probiotics, alcoholic liver disease, meta-analysis, IL-6, inflammation

## Abstract

**Background:** While probiotics may offer therapeutic benefits for alcoholic liver disease (ALD), their impact on inflammatory markers in ALD patients is still uncertain. **Objective:** This study aims to investigate the effects of probiotic supplementation on inflammatory biomarkers in patients with alcoholic liver disease, particularly examining its role in modulating interleukin-6 (IL-6) levels. **Methods:** A comprehensive search was performed across PubMed, Embase, and Web of Science to identify randomized controlled trials investigating probiotic interventions in patients with alcoholic liver disease. Seven independent comparisons were chosen for meta-analysis to evaluate probiotics’ effects on inflammatory markers, with subgroup analyses examining the impact of region, formulation type, and gender. **Results:** The findings demonstrated that probiotics led to a significant reduction in IL-6 levels (SMD = −0.68, 95% CI [−1.15; −0.20], *p* = 0.005). No statistically significant effect of probiotics on interleukin-1β (IL-1β) (SMD = −0.35, 95% CI [−0.87, 0.17], *p* = 0.18) or tumor necrosis factor alpha (TNF-α) levels (SMD = −0.73, 95% CI [−1.68, 0.21], *p* = 0.13) was observed. Notably, probiotics were associated with a significant increase in interleukin-10 (IL-10) levels (SMD = 0.93,95% CI [−0.02; 1.87], *p* = 0.05). Subgroup analyses further revealed that the efficacy of probiotics in reducing IL-6 levels was more pronounced in studies characterized by higher proportions of Asian participants, solid dosage forms, and male subjects. **Conclusions:** Probiotics have notably reduced IL-6 levels by altering the gut microbiota and increased IL-10 levels, with limited impact on IL-1β and TNF-α. These results suggest probiotics could be used to treat ALD and emphasize the need for personalized probiotic approaches for different populations.

## 1. Introduction

Alcoholic liver disease (ALD) constitutes a substantial global health burden, with its prevalence increasing in parallel with the upward trends in alcohol consumption [1]. Long-term and heavy drinking triggers inflammatory reactions in the liver, potentially resulting in various diseases such as fatty liver, liver scarring, liver cirrhosis, and liver cancer [2]. Inflammation is fundamentally linked to ALD pathogenesis, driven largely by a cascade of pro-inflammatory cytokines [3].

Recent studies highlight a strong correlation between gut microbiota and liver health [4]. An imbalance in gut microbiota can cause the intestinal barrier to become more permeable, permitting bacterial endotoxins [5], such as lipopolysaccharides (LPS), to enter the bloodstream [6]. Once in the liver, LPS interacts with Toll-like receptor 4 (TLR4), triggering the activation of Kupffer cells [7]. Once activated, these cells release reactive oxygen species (ROS), adhesion molecules, chemokines, and pro-inflammatory cytokines, leading to liver inflammation and considerable impairment of liver function [8]. Therefore, modulating the intestinal microbiota to reduce hepatic inflammation presents a promising therapeutic strategy.

Probiotics are a class of live microorganisms that, when ingested in adequate amounts, impart beneficial health effects to the host [9]. In recent years, the utilization of probiotics to enhance intestinal health has garnered considerable attention, largely attributable to their pivotal role in modulating intestinal microbiota, strengthening intestinal barrier function, and alleviating intestinal inflammation [10]. Research has indicated that probiotics may be effective in lessening inflammation in ALD [11]. However, it is important to note that much of this supporting evidence derives from preclinical models or early-phase clinical trials. Consequently, the robustness and generalizability of these findings, particularly regarding the modulation of specific cytokines in well-defined ALD patient populations, remain to be firmly established.

Mechanistically, probiotics are thought to exert their systemic anti-inflammatory effects partly through modulating the gut–liver axis [12]. By enhancing intestinal barrier integrity and shaping microbiota composition, probiotics can reduce the translocation of pathogen-associated molecular patterns [13]. This reduction subsequently dampens the activation of hepatic TLR4 and downstream signaling pathways, such as NF-κB, which are central to the transcription of pro-inflammatory cytokines, including IL-6 and TNF-α. Furthermore, probiotic-derived metabolites like short-chain fatty acids may directly influence immune cell function [14]. For instance, Lactobacillus plantarum HFY09 has been shown to help alleviate inflammation by boosting IL-10 levels and lowering pro-inflammatory factors like IL-6, IL-1β, and TNF-α [15,16]. The pathogenesis of ALD is driven by a cascade of pro-inflammatory cytokines [17,18]. The co-expression of these pro-inflammatory cytokines leads to hepatocyte dysfunction, inflammation, necrosis, and apoptosis in liver injury [19].

Building on these mechanisms, probiotics are considered a promising therapeutic approach for mitigating alcohol-induced liver inflammation through the modulation of gut microbiota [20]. However, clinical evidence regarding the efficacy of probiotics in modulating specific inflammatory cytokines in ALD patients remains inconsistent and inconclusive [21]. Existing RCTs have reported heterogeneous effects on key mediators such as IL-6 and IL-10, and comprehensive meta-analyses focusing on these cytokine-specific outcomes are lacking. These limitations include small sample sizes, divergent probiotic regimens across studies, and the absence of a comprehensive analysis targeting specific gut–liver axis cytokines. This ambiguity hinders the formulation of targeted probiotic interventions for ALD.

We hypothesized that probiotic supplementation would significantly alter the inflammatory milieu in ALD patients, specifically leading to a reduction in pro-inflammatory IL-6 and an elevation in anti-inflammatory IL-10. This study sought to comprehensively examine the ameliorative effects of probiotics on liver inflammation associated with alcoholic liver disease, focusing on the concentrations of inflammatory markers. By quantifying these pro-inflammatory and anti-inflammatory markers within the liver, the study evaluated the efficacy of probiotic therapy in preventing and reducing inflammation in alcoholic liver disease, thereby offering a scientific foundation for their clinical use.

## 2. Methods

This study was conducted in accordance with the PRISMA guidelines, and its protocol was prospectively registered with PROSPERO (Registration No. CRD420251053464).

### 2.1. Search Strategy

A systematic literature search was performed in PubMed, Embase, Web of Science, Cochrane Library, Ovid, and Sinomed, covering all records from database inception through January 2025. No language restrictions were applied at the search stage. The search strategy was designed using a combination of Medical Subject Headings (MeSH) terms and free-text keywords related to “probiotics” and “alcoholic liver disease”. The full search syntax for PubMed is provided as an example: (((((probiotics) OR (probiotic)) OR (prebiotics)) OR (prebiotic)) OR (synbiotics)) AND (((((((Fatty Liver, Alcoholic) OR (Alcoholic Fatty Liver)) OR (Alcoholic Steatohepatitis)) OR (Alcoholic liver disease)) OR (Alcoholic hepatitis)) OR (Alcoholic liver fibrosis)) OR (Alcoholic cirrhosis)). We also manually screened the reference lists of included articles and relevant reviews. Furthermore, clinical trial registries (ClinicalTrials.gov, WHO ICTRP) were searched to identify ongoing or unpublished studies.

### 2.2. Inclusion Criteria

Criteria for including and excluding inflammatory cytokine markers in ALD. Eligibility Criteria: 1. Subjects: Research including individuals diagnosed with ALD. 2. Intervention: The main therapeutic approach involves administering probiotics. 3. Comparison: Groups that receive a placebo, standard care, or other non-drug treatments. 4. Outcome Measures: Primary outcomes include levels of inflammatory cytokines. 5. Study Design: Only clinical trials will be included. 6. Data Accessibility: Studies must provide complete data and be accessible in full text. 7. Language: Only studies published in English or Chinese were included. Publication Status: Conference abstracts, unpublished data, and dissertations were excluded. Exclusion Criteria: 1. Study Type: Exclusion of non-human studies, case reports or correspondence. 2. Data Integrity: Studies were excluded if their data were insufficient, incomplete, or otherwise unavailable for analysis. 3. Comorbidities: Studies involving patients with ALD co-occurring with other chronic liver diseases will be excluded. 4. Duplicate Publications: Exclusion of studies that are duplicates or have been published multiple times. The criteria for including and excluding studies are summarized in Table 1.

These criteria are designed to ensure the quality and relevance of the included studies, providing a robust evidence base for the effects of probiotic intervention on inflammatory cytokine markers in patients with ALD.

### 2.3. Quality Assessment

The revised Cochrane Risk of Bias tool for randomized trials (RoB 2.0) was used to independently evaluate the methodological quality and potential bias of the included RCTs. This assessment was conducted by two reviewers (Jie Cui and Wenhui Li), with any discrepancies resolved through discussion or arbitration by a third reviewer. The evaluation encompassed several specific domains: the risk of bias in the randomization procedure, deviations from planned interventions, handling of missing outcome data, methods of outcome measurement, and the selection of reported results.

### 2.4. Data Synthesis and Statistical Analysis

Two independent reviewers (Jian Zhang and Ye Sun) carried out data extraction using a standardized form that had been piloted in advance. This study utilized Review Manager 5.4 and Stata 17.0. All figures were finalized and prepared for publication using Adobe Illustrator 2023. For continuous outcomes, the SMD with 95% CI was selected as the summary effect measure. The SMD was chosen over the MD because the included studies assessed the same inflammatory markers but potentially used different assay methods or units of measurement, making SMD appropriate for combining data across heterogeneous measurement scales [22]. A *p* value below 0.05 was considered statistically significant. Heterogeneity among studies was quantified using the I^2^ statistic. A fixed-effects model was applied when statistical heterogeneity was low (I^2^ < 50%); otherwise, a random-effects model was used, and potential sources of heterogeneity were explored [23,24]. Sensitivity analysis was conducted to assess the robustness of the pooled results by sequentially omitting each study [25]. Predefined subgroup analyses were planned to investigate potential sources of heterogeneity or effect modification based on the following variables: probiotic formulation, geographic region of the study population, and participant sex. These variables were selected a priori based on clinical plausibility and their potential to influence host-microbiota interactions. The evaluation of publication bias was performed primarily through visual inspection of funnel plots and Egger’s regression intercept test [26]. Additionally, to complement this assessment and adjust for potential asymmetry, the non-parametric trim-and-fill method was applied where appropriate. A *p*-value of less than 0.05 was considered indicative of potential small-study effects or publication bias.

## 3. Results

### 3.1. Study Selection and Characteristics

**Figure 1:**
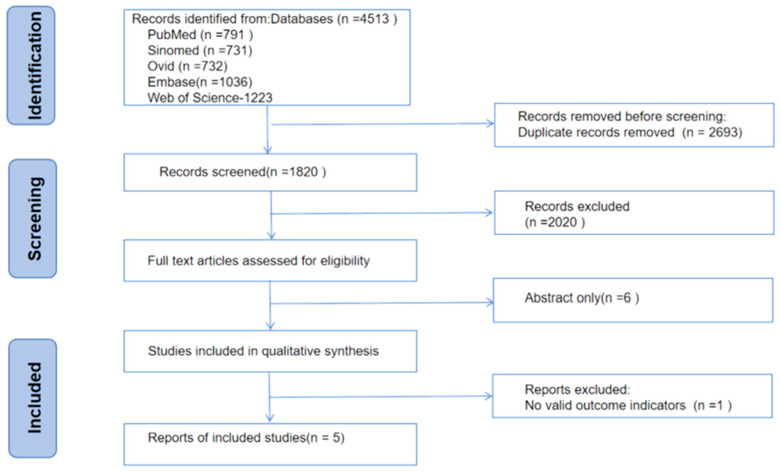
PRISMA Flowchart.

Figure 1 presents the study selection process in a PRISMA flowchart, which also details the reasons for excluding records at each stage (Figure 1). A total of 4513 studies were retrieved through the search formula: PubMed-791, Sinomed-731, Ovid-732, Web of Science-1223, and Embase-1036. Following the removal of duplicates and a review of titles and abstracts, 59 articles were identified as potentially relevant and their full texts were retrieved for detailed eligibility assessment. Five RCTs [27,28,29,30,31] met the inclusion criteria and were included in this meta-analysis. It is important to note that two of these RCTs [28,30] were multi-arm trials, each featuring two distinct probiotic intervention groups compared against a single, shared placebo control group. To adhere to meta-analytic principles and avoid unit-of-analysis error, each intervention arm was treated as an independent comparison. Consequently, the five included RCTs provided a total of seven independent comparisons for quantitative synthesis. These investigations evaluated the effects of probiotic interventions on primary inflammatory biomarkers (IL-1β, IL-6, IL-10, and/or TNF-α) in patients with ALD. Among the seven independent comparisons, data on IL-1β were available from 4 [27,28,29], IL-6 from 6 [27,28,30,31], IL-10 from 5 [27,28,31], and TNF-α from 6 [28,29,30,31].

### 3.2. Study Characteristics

The basic characteristics of the included studies are presented in Table 2. The publication years of the studies span from 2015 to 2024. A higher proportion of male participants was observed in most studies, with some studies exclusively enrolling male subjects. A variety of probiotics were administered, with intervention durations ranging from 1 to 26 weeks. The assessed outcomes included liver function parameters and inflammatory biomarkers, such as IL-1β, IL-6, IL-10, and TNF-α.

### 3.3. Risk of Bias Assessment

**Figure 2:**
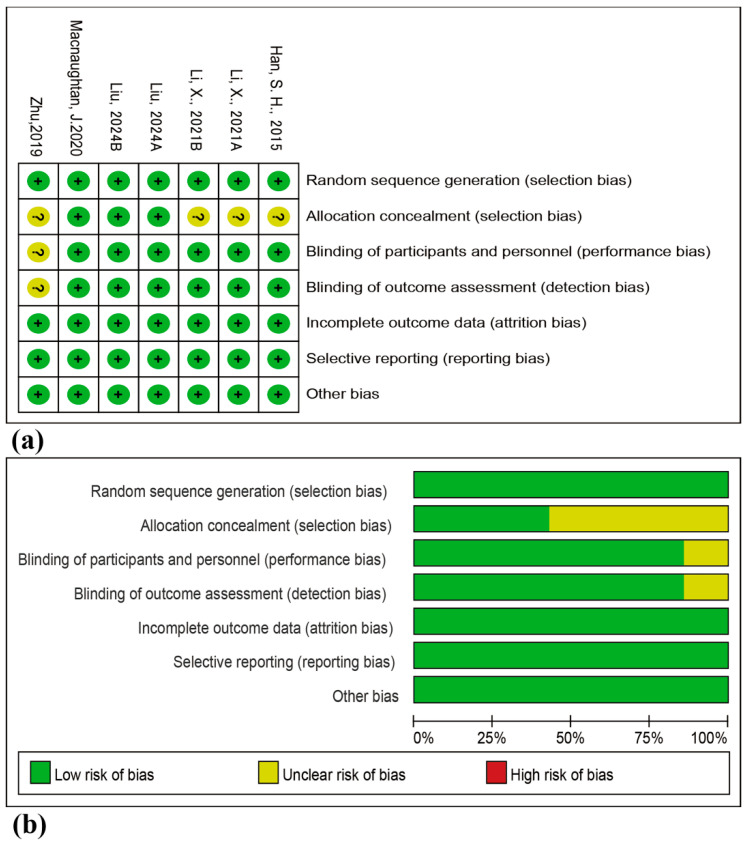
Bias Risk Assessment. (**a**) Distribution of bias across domains. (**b**) Summary of judgments: low risk (green/‘+’), some concerns (yellow/‘?’) [27,28,29,30,31].

Figure 2 summarizes the overall risk of bias assessment for the included trials (Figure 2), with detailed judgments provided in the accompanying table. Among the evaluated studies, four were rated as having a low risk of bias, while three were judged to raise some concerns.

### 3.4. Meta-Analysis and Subgroup Analysis

#### 3.4.1. Influence of Probiotics on IL-6 in ALD Patients

**Figure 3:**
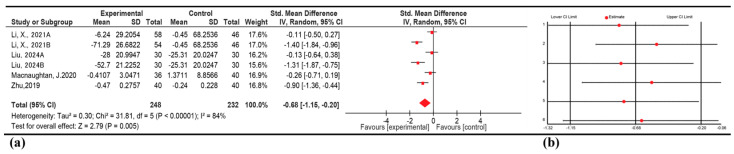
(**a**) Forest plot for the effect size and 95% confidence interval of probiotics on IL-6. (**b**) Sensitivity analysis of IL-6. Red squares indicate statistical significance (*p* < 0.05) compared to the control group [27,28,30,31].

In this analysis investigating the influence of probiotics on IL-6 levels, six studies with a total of 480 participants (248 participants were placed in the probiotic group, while 232 were in the control group) were analyzed. Given the high heterogeneity observed (I^2^ = 84%), a random-effects model was employed. The analysis showed a notable decrease in IL-6 levels among patients with alcoholic liver disease following probiotic treatment (SMD = −0.68, 95% CI [−1.15; −0.20], *p* = 0.005) (Figure 3).

**Figure 4:**
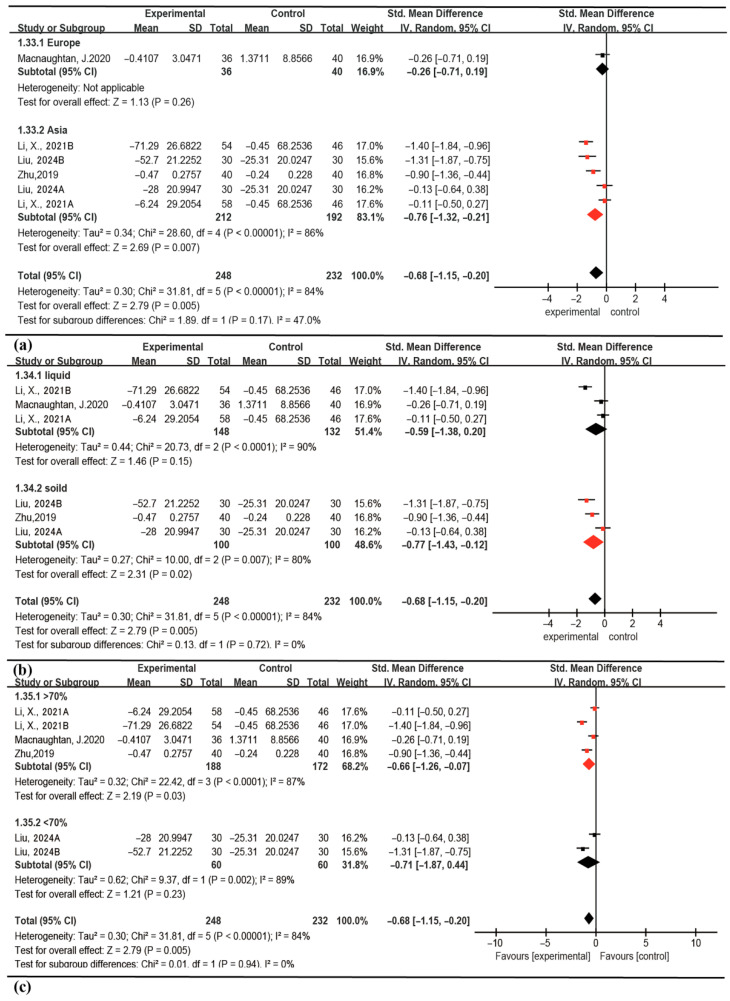
Analysis of IL-6 subgroups using Forest plots: (**a**) based on geographic region, (**b**) based on probiotic formulation, (**c**) based on gender. Red symbolRed symbols indicate subgroups with statistically significant results (*p* < 0.05) [27,28,30,31].

Exploratory subgroup analyses were conducted to investigate potential sources of the observed significant heterogeneity, focusing on factors such as geographic region, probiotic formulation, and sex distribution (Figure 4). Between-subgroup differences were observed for the Asian region subgroup (*p* = 0.007), solid-form probiotic subgroup (*p* = 0.02), and subgroups with a higher male-to-female ratio (*p* = 0.03). These exploratory findings indicate that regional, formulation, and demographic factors could be potential effect modifiers; however, this interpretation is constrained by the limited number of studies within each subgroup. Collectively, these results point to the need for future research to validate whether these variables meaningfully influence the efficacy of probiotic interventions in ALD.

#### 3.4.2. Influence of Probiotics on L-1β in ALD Patients

**Figure 5:**
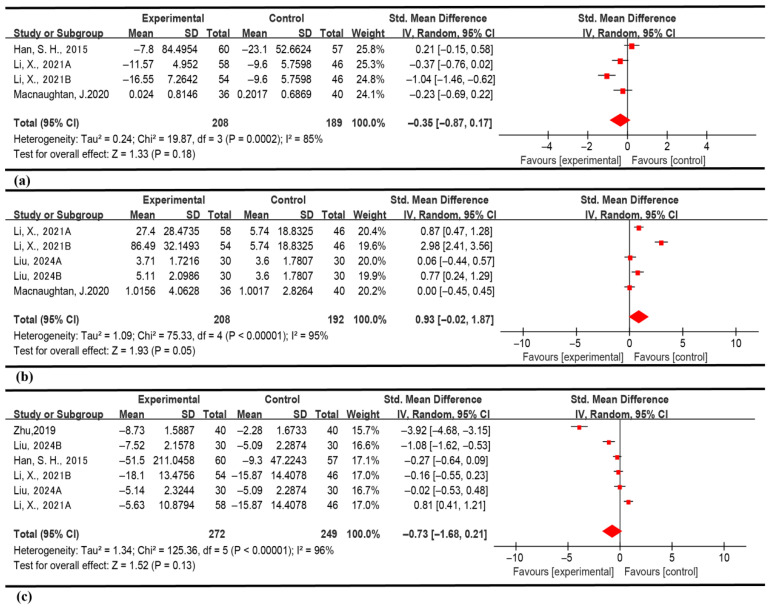
(**a**) Effect of probiotic intervention on IL-1β levels in ALD patients. (**b**) Effect of probiotic intervention on IL-10 levels in ALD patients. (**c**) Effect of probiotic intervention on TNF-α levels in ALD patients: Colors in this figure are used for visual enhancement only and do not imply statistical significance [27,28,29,30,31].

This study conducted a meta-analysis examining alterations in circulating IL-1β levels within the probiotic intervention cohort utilizing a random-effects model. The analysis incorporated four RCTs comprising 397 subjects (208 assigned to the probiotic group and 189 to the control group). The results showed that the probiotic group had a beneficial effect compared to the control group, but this effect was not statistically significant (SMD = −0.35, 95% CI [−0.87; 0.17], *p* = 0.18). This outcome suggests that probiotic supplementation does not significantly modulate systemic IL-1β concentrations (Figure 5).

From the standpoint of inflammatory regulatory mechanisms, given that IL-1β functions as a pivotal pro-inflammatory cytokine, extant experimental evidence posits that probiotics may influence macrophage IL-1β secretion via gastrointestinal microbiota modulation [28]. Nevertheless, the results of this meta-analysis reveal substantial heterogeneity in the regulatory impact of probiotics across diverse populations. Possible determinants of this heterogeneity might encompass a constrained number of clinical samples or marked variability in the duration of probiotic interventions.

#### 3.4.3. Influence of Probiotics on IL-10 in ALD Patients

In the meta-analysis examining the inflammatory factor IL-10, heterogeneity was detected (I^2^ = 95%). A statistically significant difference in IL-10 levels was observed after probiotic intervention compared with the control group (SMD = 0.93,95% CI [−0.02; 1.87], *p* = 0.05). In our study, detailed subgroup analyses were conducted for IL-10; however, no significant results were obtained. These findings further highlight the methodological constraints and challenges present in studies investigating inflammatory markers among individuals with alcoholic liver disease (Figure 5).

#### 3.4.4. Influence of Probiotics on TNF-α in ALD Patients

This meta-analysis evaluated the impact of probiotic supplementation on TNF-α levels in patients diagnosed with ALD. The analysis included six randomized controlled trials comprising a total of 521 participants (272 receiving probiotics and 249 in the control group). Due to the substantial heterogeneity among the studies (I^2^ = 96%), a random-effects model was employed. The analysis demonstrated no significant effect of probiotic intervention on TNF-α levels (SMD = −0.73, 95% CI [−1.68; 0.21], *p* = 0.13). Previous research has indicated that probiotics may exert a regulatory influence on TNF-α in patients with alcoholic liver disease [29]. However, this meta-analysis failed to achieve statistical significance. This discrepancy may be attributed to the marked variability in the inhibitory effects of different probiotic strains on TNF-α. Specifically, Lactobacillus rhamnosus has been shown to exhibit a less pronounced regulatory effect on TNF-α [30] (Figure 5).

### 3.5. Assessment of Publication Bias

Visual inspection of the funnel plot suggested symmetry, and the Egger test (*p* = 0.442) indicated no significant publication bias in the meta-analysis (Figure S1).

## 4. Discussion

Our meta-analysis demonstrated that probiotic intervention significantly reduced IL-6 levels while elevating IL-10 in ALD patients, with no significant impact on IL-1β or TNF-α [11,32,33]. This selective regulatory pattern suggests that probiotics may affect hepatic inflammatory networks through specific immunomodulatory mechanisms [34]. While the included clinical trials did not assess underlying mechanisms, the observed cytokine profile is consistent with preclinical evidence suggesting that probiotic-mediated remodeling of the gut microbiota [35], particularly through metabolites like short-chain fatty acids (SCFAs), may regulate Kupffer cell polarization via the gut–liver axis [36]. It is plausible that this modulation suppresses the TLR4/MyD88 signaling pathway, leading to decreased release of IL-6 [37]. Similarly, the increase in IL-10 could be linked to the potential enhancement of regulatory T cell (Treg) differentiation [38] and subsequent STAT3 signaling [39]. This study underscores the crucial impact of probiotics on liver inflammation through gut–liver axis interactions and specific immune regulation. It provides a foundational framework for further elucidation of the mechanistic pathways involved in ALD.

Notably, the lack of significant effect on IL-1β and TNF-α might be explained by the possibility that their production is governed by pathways less amenable to probiotic modulation within the timeframe and design of the included studies. For instance, the sustained activation of the NLRP3 inflammasome, a key driver of IL-1β maturation in ALD [40]. Acetaldehyde, a primary metabolite of alcohol, can activate this inflammasome [41], creating a persistent inflammatory loop [42] that may be relatively refractory to the primary actions of conventional probiotics on gut barrier function and microbiota composition [43,44,45]. Likewise, TNF-α expression is tightly regulated by the NF-κB pathway [46,47], which might require more direct or prolonged intervention to be significantly altered [48,49].

To explore potential sources of the observed heterogeneity, we performed subgroup analyses based on formulation, geographic region, and patient gender, which indicated potential variations in efficacy associated with these factors. These effects may be linked to the regulation of the gut–liver axis, microbial metabolite synthesis, and modulation of the host immune microenvironment. These preliminary observations serve to generate specific, mechanistic hypotheses for future research. For example, the apparent superior effect of solid formulations (e.g., enteric-coated tablets) could be explained by their enhanced ability to ensure higher colonic delivery of viable bacteria [50]. This, in turn, may promote the sustained production of bioactive metabolites like butyrate [51,52,53], which are hypothesized to modulate hepatic inflammation through pathways such as AMPK-dependent inhibition of TLR4/MyD88 and NF-κB [54]. In contrast, the lower gastric survival of liquid formulations might result in insufficient colonic metabolite production to exert sustained effects [55].

Similarly, analyses indicated that probiotics had a more significant impact on reducing IL-6 levels in Asian populations, while studies involving European groups did not show statistically significant results [56]. This discrepancy may stem from fundamental host-microbiota metabolic interdependencies. The greater reduction in IL-6 observed in Asian populations could be associated with enterotype characteristics and impaired butyrate production in alcohol-damaged intestines [57], which probiotics might compensate for [58]. Conversely, the attenuated response in European populations may reflect diet-induced modulation of the bile acid pool, as Western high-fat diets induce FXR signaling, which competes with probiotic-derived secondary bile acids for nuclear receptor activation [59,60]. Meanwhile, the high intake of fermented foods in traditional Asian diets may pre-adapt the gut environment, enhancing probiotic colonization [61]. The gut microenvironment in Asian populations may be more conducive to probiotic growth and proliferation, thereby augmenting their IL-6 ameliorative effects [62].

**Figure 6:**
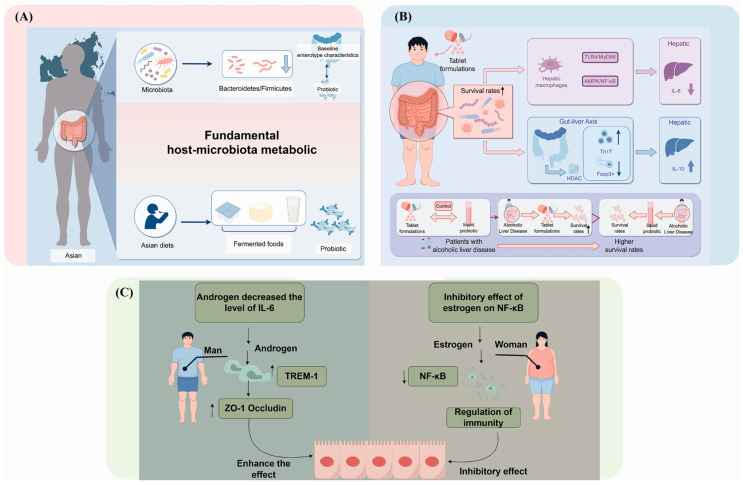
Schematic summary of proposed mechanisms underlying subgroup disparity: (**A**) Microbiota-related mechanisms: Differences in gut microbiota composition and host–microbiota metabolic interactions are influenced by Asian diets, fermented foods, and probiotic intake, contributing to subgroup heterogeneity. (**B**) Gut–liver axis and immune regulation: Probiotic tablet formulations enhance survival rates and modulate hepatic macrophages via pathways such as TURACOM, AMPK/NF-κB, and HDAC. These effects regulate the gut–liver axis, Th17/Foxp3+ balance, and IL-10 production, thereby influencing ALD outcomes and patient survival. (**C**) Sex hormone-related mechanisms: Androgen decreases IL-6 levels via TREM-1 and upregulates tight junction proteins (ZO-1, occludin), enhancing barrier function. Estrogen exerts an inhibitory effect on NF-κB, contributing to immune regulation and sex-based differences in ALD susceptibility and progression. Abbreviations: ALD: alcoholic liver disease; AMPK: AMP-activated protein kinase; HDAC: histone deacetylase; IL: interleukin; NF-κB: nuclear factor kappa-B; Th17: T helper 17 cells; Foxp3+: forkhead box P3-positive regulatory T cells; TREM-1: triggering receptor expressed on myeloid cells 1; ZO-1 zonula occludens-1.

We observed that the efficacy of probiotics was clearly influenced by gender [63]. In male subjects, IL-6 levels were reduced by 37.5%. Previous studies have elucidated the complex interplay between androgens, TREM-1, inflammation, immune regulation, and gender differences [64]. Androgens may modulate the expression and function of inflammatory pathways, thereby influencing individual responses to inflammatory stimuli and sensitivity to immune-modulatory therapies, potentially conferring greater benefits from probiotic immunomodulation in male patients with alcoholic liver disease [65,66]. Estrogen-mediated inhibition of NF-κB in females, conversely, might introduce a ceiling effect that masks additional probiotic benefits [67,68]. Figure 6 provides an integrative summary of the potential mechanisms that may underlie the observed subgroup disparities (Figure 6). These proposed pathways, formulated at the intersection of our clinical findings and established biological knowledge, highlight promising avenues for further investigation to elucidate the personalized effects of probiotics.

This study systematically elucidated the heterogeneity characteristics and molecular mechanisms underlying probiotic-mediated modulation of inflammatory responses in ALD, thereby establishing a theoretical framework for personalized strain selection and formulation development. However, it must be acknowledged that current meta-analyses are constrained by an insufficient number of RCTs, a lack of long-term follow-up data, and substantial statistical heterogeneity attributable to variations in study design. To bridge these gaps and advance toward precision therapy, future research should follow a progressive pathway. The immediate priority is to conduct large-scale, well-designed RCTs to prospectively validate the key variables identified here, such as probiotic formulation, geographic origin, and host sex. Subsequently, to decipher the mechanisms behind these effects, studies should integrate multi-omics analyses (e.g., metagenomics, metabolomics) with clinical outcomes to delineate how specific probiotic strains and host factors interact to shape the immune response. Building on this mechanistic understanding, predictive modeling and machine learning can then be employed to optimize strain selection and personalize intervention strategies. These findings provide a roadmap for future research, moving from large-scale clinical validation to mechanistic studies, and ultimately to the development of personalized probiotic therapies for ALD.

## 5. Conclusions

This meta-analysis offers a comprehensive evaluation of the effects of probiotics on inflammatory markers in individuals with ALD, revealing significant modulatory effects on specific cytokines. Pooled data indicate a statistically significant reduction in IL-6 levels (SMD = −0.68, 95% CI [−1.15; −0.20]) and an increase in IL-10 levels (SMD = 0.93, 95% CI [−0.02; 1.87]), while effects on IL-1β and TNF-α were not significant. These results suggest that probiotics can modulate the gut–liver axis to mitigate inflammation in ALD, potentially through the regulation of gut microbiota and enhancement of intestinal barrier function.

However, these results should be interpreted considering the limitations of the included studies and this meta-analysis, such as substantial statistical heterogeneity (I^2^ > 80%), a limited number of RCTs, and variations in probiotic strains and protocols.

Subgroup analyses revealed that the efficacy of probiotics in reducing IL-6 levels was influenced by regional, formulation, and demographic factors. Specifically, probiotics were more effective in Asian populations, solid dosage forms, and male subjects. These findings underscore the importance of personalized probiotic interventions tailored to specific populations and formulations. However, these subgroup findings are exploratory and hypothesis-generating due to the limited number of studies within each comparison and require validation in future prospective trials.

To translate these findings towards clinical application, future research must prioritize large-scale, rigorous RCTs and integrated mechanistic studies to develop evidence-based, personalized probiotic strategies.

In conclusion, probiotics offer a promising therapeutic approach for modulating inflammation in ALD, particularly by reducing IL-6 and enhancing IL-10. Further high-quality research is essential to define their precise role within the therapeutic arsenal for ALD.

## Figures and Tables

**Table 1:** PICOS criteria for determining inclusion and exclusion.

Parameters	Inclusion and Exclusion Criteria
Participants	The study involves patients with ALD, including alcoholic fatty liver, alcoholic hepatitis, alcoholic liver fibrosis, and alcoholic cirrhosis.
Intervention	The main treatment approach is the administration of probiotics.
Comparison	Control groups receive a placebo, standard care, or other non-pharmacological treatments.
Outcomes	Main results include the concentrations of inflammatory cytokines such as IL-1β, IL-6, IL-10, or TNF-α.
Study design	RCT studies.

**Table 2:** Features of the research.

Author (Year)	Location	Sample Size (I/C)	Age (I/C) (Years)	Probiotics	Comparison	Duration (Week)	Outcomes	Level of Disease	Male	Form of Intervention	Dose
Macnaughtan, J. et al. (2020) [27]	UK	36/40	56.16 ± 8.47/58.16 ± 9.18	*Lactobacillus casei*	Placebo	26	IL-1β, IL-6, IL-10	ALC	71%	Liquid	1 × 10^8^ CFU/mL
Li, X. et al. (2021) A [28]	China	58/46	51.10 ± 3.90/52.60 ± 5.67	*Lactobacillus casei*	Placebo	8	TNF-α, IL-1β, IL-6, IL-10	AFL	100%	Liquid	2 × 10^8^ CFU/mL
Li, X. et al. (2021) B [28]	China	54/46	49.6 ± 4.17/52.60 ± 5.67	*Lactobacillus casei*	Placebo	8	TNF-α, IL-1β, IL-6, IL-10	AFL	100%	Liquid	1 × 10^8^ CFU/mL
Han, S. H. et al. (2015) [29]	South Korea	60/57	52.7 ± 11.3	*Lactobacillus subtilis. Streptococcus faecium*	Placebo	1	TNF-α, IL-1β	AH	64%	Solid Dosage Forms	1200 mg/g
Liu et al. (2024) A [30]	China	30/30	51.92 ± 10.02/52.31 ± 9.08	*Clostridium casei*	Placebo	13	TNF-α, IL-6, IL-10	ALC	67%	Tablets	1.75 × 10^7^ CFU/g
Liu et al. (2021) B [30]	China	30/30	52.58 ± 10.37/52.31 ± 9.08	*Clostridium casei*	Placebo	13	TNF-α, IL-6, IL-10	ALC	68%	Tablets	1.75 × 10^7^ CFU/g
Zhu et al. (2019) [31]	China	40/40	47.2 ± 5.1/46.9 ± 5.2	*Clostridium casei*	Placebo	8	IL-6, TNF-α	--	83%	Tablets	1 × 10^6^ CFU/g

## Data Availability

No new data were created or analyzed in this study. Data sharing is not applicable to this article.

## Supplementary Materials

The following supporting information can be downloaded at: https://www.mdpi.com/article/10.3390/nu18040666/s1, Table S1: PRISMA Checklist; Figure S1: Assessment of Publication Bi.
