# Supplementary material for: Evaluating the Efficacy of Probiotics on Inflammatory Cytokines in Alcoholic Liver Disease: A Focus on IL-6 and IL-10
Source: Nutrients. 2026 Feb 18;18(4):666. doi: 10.3390/nu18040666 (PMC12943775; doi:10.3390/nu18040666)
Supplement: Supplementary file 1 [file nutrients-18-00666-s001.zip › Table S1 PRISMA Checklist.pdf]

Evaluating the Efficacy of Probiotics on Inflammatory Cytokines in Alcoholic Liver Disease: A Focus on IL-6 and IL-10

Jiadila Bahetiyaer [1,2], Jie Cui [3], Wenhui Li [3], Jian Zhang [3], Ye Sun [3], Chunqing Ai [1], Shuying Liu [1], Shuaiming Jiang [4,*], Chengcheng Zhang [5,*] and Jinchi Jiang [1,3,*]

1 State Key Laboratory of Marine Food Processing & Safety Control, Qingdao 266400, China; 17366104413@163.com (J.B.); acqdongying@163.com (C.A.); shuying.liu@bmsg.com (S.L.)

2 College of Biological and Pharmaceutical Engineering, Nanjing Tech University, Nanjing 211816, China

3 College of Food Science and Light Industry, Nanjing Tech University, Nanjing 211816, China; jiecui_nitech@163.com (J.C.); lwhnjtec@163.com (W.L.); four-leaf-clover@njtech.edu.cn (J.Z.); sunye@nitech.edu.cn (Y.S.)

4 Key Laboratory of Food Nutrition and Functional Food of Hainan Province, School of Food Science and Engineering, Hainan University, Haikou 570228, China

5 State Key Laboratory of Food Science and Resources, School of Food Science and Technology, Jiangnan University, Wuxi 214122, China

* Correspondence: shuaimingjiang@hainanu.edu.cn (S.J.); zhangchengcheng@jiangnan.edu.cn (C.Z.); jiangjinchi@126.com (J.J.)

Supplementary Materials

1.1 Table S1 PRISMA Checklist

Section/topic	#	Checklist item	Reported on page #
**TITLE**			
Title	1	Identify the report as a systematic review, meta-analysis, or both.	Page 1
**ABSTRACT**			
Structured summary	2	Provide a structured summary including, as applicable: background; objectives; data sources; study eligibility criteria, participants, and interventions; study appraisal and synthesis methods; results; limitations; conclusions and implications of key findings; systematic review registration number.	Page 1
**INTRODUCTION**			
Rationale	3	Describe the rationale for the review in the context of what is already known.	Page 1-2
Objectives	4	Provide an explicit statement of questions being addressed with reference to participants, interventions, comparisons, outcomes, and study design (PICOS).	Page 2
**METHODS**			
Protocol and registration	5	Indicate if a review protocol exists, if and where it can be accessed (e.g., Web address), and, if available, provide registration information including registration number.	Page2
Eligibility criteria	6	Specify study characteristics (e.g., PICOS, length of follow-up) and report characteristics (e.g., years considered, language, publication status) used as criteria for eligibility, giving rationale.	Page 3
Information sources	7	Describe all information sources (e.g., databases with dates of coverage, contact with study authors to identify additional studies) in the search and date last searched.	Page 2
Search	8	Present full electronic search strategy for at least one database, including any limits used, such that it could be repeated.	Page 2
Study selection	9	State the process for selecting studies (i.e., screening, eligibility, included in systematic review, and, if applicable, included in the meta-analysis).	Page 4
Data collection process	10	Describe method of data extraction from reports (e.g., piloted forms, independently, in duplicate) and any processes for obtaining and confirming data from investigators.	Page 5

Data items	11	List and define all variables for which data were sought (e.g., PICOS, funding sources) and any assumptions and simplifications made.	Page 5
Risk of bias in individual studies	12	Describe methods used for assessing risk of bias of individual studies (including specification of whether this was done at the study or outcome level), and how this information is to be used in any data synthesis.	Page 6
Summary measures	13	State the principal summary measures (e.g., risk ratio, difference in means).	Page 3
Synthesis of results	14	Describe the methods of handling data and combining results of studies, if done, including measures of consistency (e.g., I^2) for each meta-analysis.	Page 3

Section/topic	#	Checklist item	Reported on page #
Risk of bias across studies	15	Specify any assessment of risk of bias that may affect the cumulative evidence (e.g., publication bias, selective reporting within studies).	Page 6
**RESULTS**			
Study selection	16	Give numbers of studies screened, assessed for eligibility, and included in the review, with reasons for exclusions at each stage, ideally with a flow diagram.	Page 4
Study characteristics	17	For each study, present characteristics for which data were extracted (e.g., study size, PICOS, follow-up period) and provide the citations.	Page 5
Risk of bias within studies	18	Present data on risk of bias of each study and, if available, any outcome level assessment (see item 12).	Page 6
Results of individual studies	19	For all outcomes considered (benefits or harms), present, for each study: (a) simple summary data for each intervention group (b) effect estimates and confidence intervals, ideally with a forest plot.	Page 6-10
Synthesis of results	20	Present results of each meta-analysis done, including confidence intervals and measures of consistency.	Page 6-10
Risk of bias across studies	21	Present results of any assessment of risk of bias across studies (see Item 15).	Page 6
**DISCUSSION**			
Summary of evidence	22	Summarize the main findings including the strength of evidence for each main outcome; consider their relevance to key groups (e.g., healthcare providers, users, and policy makers).	Page 11-14
Limitations	23	Discuss limitations at study and outcome level (e.g., risk of bias), and at review-level (e.g., incomplete retrieval of identified research, reporting bias).	Page 14
Conclusions	24	Provide a general interpretation of the results in the context of other evidence, and implications for future research.	Page 13-14
**FUNDING**			
Funding	25	Describe sources of funding for the systematic review and other support (e.g., supply of data); role of funders for the systematic review.	Page 14

For more information, visit: **www.prisma-statement.org**.
