# Supplementary figures and images for: Evaluating the Efficacy of Probiotics on Inflammatory Cytokines in Alcoholic Liver Disease: A Focus on IL-6 and IL-10
Source: Nutrients. 2026 Feb 18;18(4):666. doi: 10.3390/nu18040666 (PMC12943775; doi:10.3390/nu18040666)

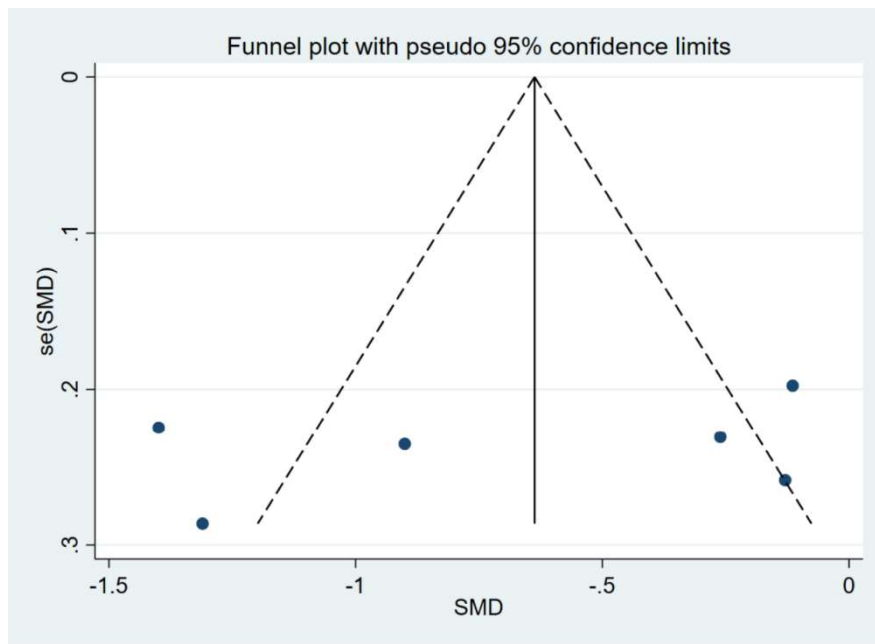

(a)

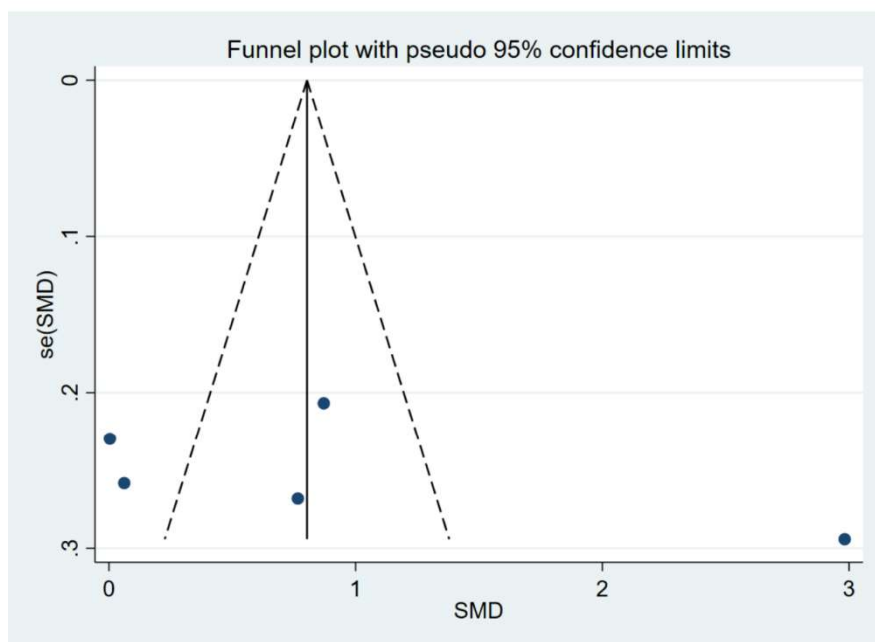

(b)

**Figure S1.** Assessment of Publication Bi

Supplement: Supplementary file 1 [file nutrients-18-00666-s001.zip › Supplementary figure.pdf]
